# Annotations

**Published:** 1894-12-01

**Authors:** 


### Annotations.

### The Treatment of Boils.

It is desirable to have other resources than the knife and the antiseptic lotion in the treatment of boils, inasmuch as the mere mention of the lancet is painfully distressing to some over sensitive natures. Van Hoorn employs a method which fulfils the threefold condition of being antiseptic, comparatively painless, and said to be very effectual. He first makes the patient's body aseptic by washing the whole of it quite clean with tepid water and potash soap. He then proceeds to a still more perfect purification of the boil and the surrounding inflamed parts by laving them thoroughly with corrosive sublimate solution of the strength of one in 1,000. Here perhaps we may interrupt our description by the suggestion that a solution of the biniodide of mercury, which, according to Bolsheolsky, Luff, Illingworth, and others is a safer and more powerful germicide than the perchloride, might be employed instead. Having made the outer parts thoroughly aseptic, Van Hoorn completely covers the furuncle and its immediately surrounding area with a phenol and mercurial plaster. This he changes daily. Of course it may be changed oftener if necessary. If the furuncle have reached the definitely purulent stage it bursts. The contents are then thoroughly squeezed out and the cavity cleansed with the sublimate solution. It sometimes happens, however, that treatment is commenced before fluctuation has declared itself, and in that case absorption and abortion may be looked for. It is said that the results obtained are excellent. The inflamed parts quickly return to the normal state, cicatrisation is rapid, and the cure is both pleasant and prompt. It would be well that this method of treatment should be extensively tried. If it should prove as effectual in other hands as in those of Van Hoorn it will be a distinct addition to the resource of general practice.

### The Great Northern Central Hospital.

The controversy between the general practitioners of the North of London and the authorities of the Great Northern Central Hospital on the question of the opening of pay wards at that institution is becoming somewhat embittered, and threatens to be prolonged. The history of the hospital does not date very far back, and the principles on which it was, originally at least designed to be established are well known to a good many persons in the hospital world. When the movement for a new hospital was initiated in North London one of the objects aimed at was the better instruction of the general practitioners of the northern quarter. The original movers were anxious to found an institution which should not only treat poor patients, but should be carried out on such modern lines as would make it thoroughly popular with local medical men and afford them a much needed rallying point and centre of post-graduate stimulus and study. That in the estimation of local practitioners the hospital has not as yet been a help to them in any of these directions it need not be doubted; that in future its presence among them will be prejudicial to their interests in other directions they themselves honestly fear. Under these circumstances the committee may well make a public-spirited effort to reconsider a good many questions from the standpoint of the local practitioners, as well as from the points of view of the patients, the honorary medical staff, and the general public. One grievance stands out in specially high relief before the minds of the general practitioners in the district. The original promoters of the hospital proposed that the pay wards should be occupied by a class of patients, such as clerks, shop assistants, and other single persons living in lodgings who could afford to pay medical fees, and who, therefore, were to be entitled to have the attendance of their own medical men in the hospital pay wards. That part of the scheme has been dropped or forgotten. Local medical practitioners are not to be permitted to follow their patients into the pay wards. This is felt to be the greatest of all the grievances of which complaint is made against the hospital authorities. Is this permission to be granted? And if it is not, why is it not? The local doctors are certainly entitled to press these questions home on the hospital authorities; and we cannot but hope that the governing body will prove itself capable of taking a broad, enlightened, and modern view of their duty, and will have the courage to stand firmly by their convictions. New times and new circumstances sometimes demand the modification of even the most honoured routine usages, alike in hospitals as in general affairs.

### The Possibilities of Cerebral Surgery.

An extremely interesting case of subcortical cerebral tumour treated by operation was shown at the Medical Society last Monday by Dr. Beevor and Mr. Ballance, illustrating very well the accuracy of diagnosis and the radical nature of the treatment, which recent physiology and modern surgery render possible. It was pointed out in the discussion that out of a large number of cases of cerebral tumour examined in the post-mortem room only a small proportion, about 10 per cent., were so situated as to appear capable of removal. In reply to this, however, it was stated that cases of tumour in the cerebellum are not so absolutely beyond the pale as has been supposed, and it was also shown that operation, even if it did not eventuate in removal of the growth, might and did give great relief to the symptoms which, in many cases of intracranial tumour, rendered life almost unendurable. Although removal of the tumour is the thing most to be desired, still the distressing symptoms, the agonizing pain, the sickness, the vertigo, and the mental enfeeblement arise from its pressure rather than from its presence, and this may be to a large extent relieved by an operation involving division of the dura mater, even though the tumour itself be not removed nor even found. This is important. The later days of many patients suffering from cerebral tumour are so full of misery that it is something to feel that, even if on opening the cranium it should not be possible to remove the tumour, at least some relief can be fairly hoped for from the more distressing symptoms during such time as life may last.

